# Calibration and sensitivity enhancement of AI rate integrator through EKF and impact on raman spectroscopy optimal wavelengths

**DOI:** 10.1371/journal.pone.0351113

**Published:** 2026-06-24

**Authors:** Jafar Keighobadi, Farnaz Imani

**Affiliations:** Faculty of Mechanical Engineering, University of Tabriz, Tabriz, East Azerbaijan, Islamic Republic of Iran; University of Tabriz, CHINA

## Abstract

Developments in atomic rate sensors have opened new opportunities for achieving high-precision rate integration. This study investigates the measurement principles and calibration parameters of an atomic interferometry (AI) rotation-rate sensor, modeled through Raman beam interactions and validated using real trajectory data. The primary objective is to identify and estimate the sensor parameters in order to improve orientation accuracy. To this end, real test data were collected from GPS-aided inertial systems (AIDIS 16407 and Vitans) along a predefined route. These data were incorporated into a software-in-the-loop AI gyroscope model, in which Raman laser beams acted as the excitation signal. A two-stage cascaded Extended Kalman Filter (EKF) was developed to simultaneously estimate sensor bias and calibration parameters. This approach enables parameter identification without requiring a physical AI gyroscope, thereby providing a cost-effective calibration framework. The experimental results demonstrate a significant reduction in bias uncertainty from 10^−6^ to 10^−10^ rad/s, along with a 35% improvement in orientation accuracy compared to the uncalibrated case. Furthermore, the calibrated AI parameters were applied to Raman spectroscopy of Rubidium (Rb), where an optimal wavelength of 1100 nm increased the Raman peak intensity by 20%, enhancing molecular bond resolution and spectral clarity.These findings confirm that the proposed calibration approach not only improves navigation accuracy but also enhances Raman spectroscopic analysis, highlighting the interdisciplinary potential of AI-based sensors.

## Introduction

Reliable and high-precision navigation capability is a key requirement for modern autonomous and guided systems, spanning applications from intelligent transportation and aerospace missions to marine navigation and planetary exploration [1–3]. In such scenarios, navigation states are often estimated independently of external references, relying solely on inertial measurements. However, conventional inertial navigation systems suffer from cumulative errors caused by sensor bias and noise. These errors lead to rapid drift due to the double and triple integration of acceleration and angular rate measurements, significantly limiting long-term accuracy and reliability [4].

Accurate vehicle positioning and orientation determination rely on Newton’s laws of motion and measurements obtained from accelerometers and angular rate sensors [5]. These instruments must provide sufficient information to capture specific forces in a three-dimensional reference frame. Since second- and third-order integrations of specific force and angular rate measurements are required to estimate velocity, position, and attitude, the resulting solutions are highly susceptible to rapid drift over time due to sensor imperfections [5–7].

Due to the high precision of atomic rate sensors and matter-wave accelerometers deployed on moving platforms, remarkable performance has been demonstrated in both aerial and marine environments [8]. High-sensitivity, low-drift inertial sensors based on atom interferometry are expected to significantly advance inertial guidance and navigation systems [9,10]. One of the key approaches to further improving the performance of AI rate sensors is the systematic investigation of their error sources [11]. smaller than those of conventional dynamically tuned and navigation-grade inertial navigation systems (INS). This substantial improvement in accuracy motivates the integration of such high-precision inertial sensors with complementary sensing modalities. However, several challenges remain before these systems can be reliably employed for practical navigation applications [12]. Raman pulse atom interferometry has been successfully applied in numerous high-precision inertial sensing studies, as reported in [13–19].

Quantum inertial sensors based on matter-wave and atom interferometry have emerged as transformative technologies due to their ultra-low bias drift, high sensitivity, and long-term measurement stability [20–22]. Both experimental and theoretical studies have demonstrated that cold-atom gyroscopes and accelerometers can achieve precision levels several orders of magnitude higher than those of classical inertial sensors, particularly in long-duration and drift-sensitive applications [23]. Moreover, advanced control strategies and hybrid architectures that integrate atom-interferometric sensors with classical inertial measurement units (IMUs) have been shown to improve robustness against environmental perturbations and platform dynamics, thereby enhancing operational feasibility under real-world conditions [3,23–25].

Despite these promising developments, several critical challenges remain unresolved. In particular, robust online calibration methods capable of simultaneously estimating sensor biases and calibration parameters under dynamic, real-world conditions are still lacking [26]. In addition, the effective integration of physics-based quantum sensor models into navigation filters, while preserving computational efficiency and robustness, remains an open research problem. Moreover, many existing studies rely on idealized simulations or require complex and costly experimental setups, which limits their practical applicability in real-world navigation systems [20].

In this paper, we present a comprehensive framework for the modeling, calibration, and performance evaluation of an AI-based rate sensor. The study is motivated by the need to improve angular rate and orientation estimation using atom interferometry. A novel AI rate sensor configuration is introduced, and its key parameters are estimated using an Extended Kalman Filter (EKF). The proposed approach enables precise calibration and alignment, leading to enhanced accuracy in attitude and heading estimation, as well as overall orientation determination.

To verify the sensitivity of the AI rate integrator and enhance the identification of the Rb spectrum, an additional experimental study is conducted. In this experiment, a Raman laser generator is employed to produce a two-pulse laser beam, which serves as the actuation input to the AI rate integrator model.

Due to the practical limitations associated with accessing a physical AI rate integrator, high-precision data from a conventional inertial gyroscope are used as the input source. These data are further corrected using GPS measurements within a coupled INS/GPS framework and are treated as reference outputs for the designed AI angular rate sensor. By combining the Raman laser inputs with the corrected INS/GPS outputs, a complete input–output dataset is constructed for the identification and estimation of the AI rate integrator calibration and alignment parameters, as well as the precise wavelength λ.

The results demonstrate the high sensitivity of the proposed AI-based system and its capability to enhance spectroscopic analysis. The proposed approach enables the simultaneous estimation of calibration parameters and sensor bias without requiring a physical atomic gyroscope, providing a practical and cost-effective solution. This framework improves inertial navigation accuracy and robustness, and facilitates the integration of atomic inertial sensors into real-world navigation applications, while also supporting precise atomic-scale measurements.

## Materials and methods

### Basics of atom interferometry and phase modeling

#### Physical principle of raman atom interferometry.

Atom interferometry exploits the wave–particle duality of matter by coherently manipulating atomic de Broglie waves to generate interference patterns. In Raman atom interferometers, stimulated Raman transitions are used to coherently couple two hyperfine ground states of an atom via a pair of phase-locked laser beams [27,28]. This interaction enables precise control of both the atomic momentum and internal states, allowing the implementation of beam splitters and mirrors for matter waves [29].

A Mach–Zehnder atom interferometer (MZAI) is typically realized using a sequence of three Raman light pulses (π/2–π–π/2), which split, redirect, and recombine atomic wave packets along two distinct interferometric paths. The phase difference accumulated between these paths encodes information about inertial quantities such as acceleration and rotation.

Owing to their ultra-low drift and high sensitivity, Raman-based atom interferometers have emerged as a promising technology for next-generation inertial sensing and navigation systems [28].

Cold Rb atoms are considered due to their widespread use in atomic interferometry applications. To implement atom interferometry, sequences of light pulses are applied to drive Raman transitions. In this configuration, the Raman transition is a two-photon process that coherently couples the two hyperfine ground states of an Rb atom via a virtual excited state rather than a real one.

This process involves the absorption of a photon from one laser beam followed by stimulated emission into a second beam, enabling precise control of both the internal states and momentum of the atoms. Such two-photon virtual-level transitions are widely employed in AI systems, ensuring accurate manipulation of atomic populations for high-precision interferometric measurements.

**Fig 1:**
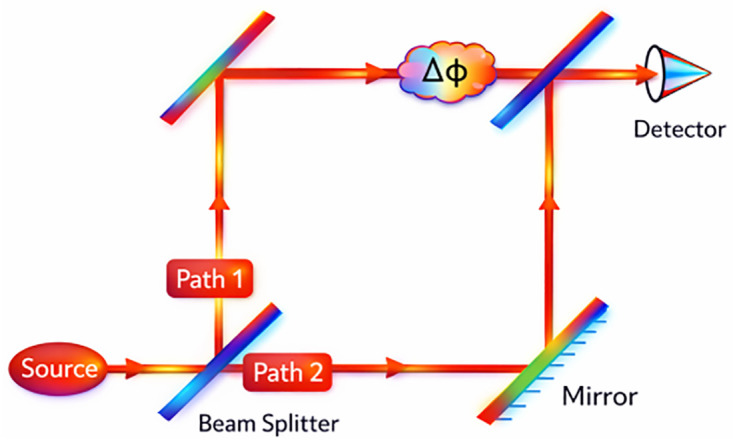
A scheme of atom interferometer in MZAI configuration.

In the MZAI, as illustrated in Fig 1, the two trajectories of the atomic wave packet are formed by applying a sequence of Raman laser pulses to a cloud of cold atoms. Each Raman pulse drives a stimulated two-photon transition between two hyperfine ground states via a far-detuned virtual excited state. This coherent interaction splits the atomic wave packet into two distinct paths separated in both space and time.

The two paths subsequently recombine, producing an interference signal. Unlike spontaneous Raman scattering, this process enables precise control of the atomic momentum and phase. As a result, the MZAI geometry can be accurately implemented, enabling high-precision inertial measurements.

**Fig 2:**
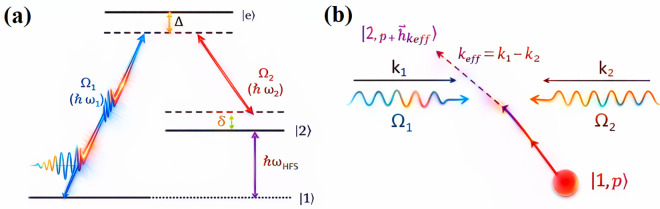
Energy level diagram of an effective two-level Raman transition in a Rubidium (Rb) atom: (a) co-propagating Raman transition, and (b) counter-propagating Raman transition.

The states |1〉 and |2〉 in Fig 2 correspond to the hyperfine ground states|5S^1/2^, F = 3〉 and |5S^1/2^, F = 4〉 of Rb, respectively, separated by the hyperfine splitting (HFS) energy ${\text{ħ}\omega }_{HFS}$. The state |e〉 represents a far-detuned virtual excited state associated with the |4P^3/2^, F_0_ = 2〉 level and is not significantly populated during the Raman transition. In our cold atom interferometer, we use a two-photon Raman transition to couple the hyperfine ground states ∣1⟩ and ∣2⟩ of Rb via a far-detuned virtual excited state ∣e⟩. The virtual state ∣e⟩ is not significantly populated during the transition.

Quantum mechanical systems governed by the Schrödinger equation are commonly described within the framework of plane-wave interferometry theory [30]. Such systems include controlled manipulation of molecular wave packets [31–33], high-harmonic generation processes [34], and the implementation of quantum computing algorithms [35].

#### Parametric quantum model of atom–light interaction.

The coherent interaction between an atomic ensemble and Raman laser fields is described within the framework of non-relativistic quantum mechanics using the time-dependent Schrödinger equation [36] . In this formulation, the system dynamics are governed by a parameter-dependent Hamiltonian H(θ), which incorporates both the intrinsic atomic energy structure and the effective coupling induced by Raman transitions. The quantum state of the system is represented by the time-dependent wavefunction Ψ(t) in the corresponding Hilbert space. Accordingly, the system evolution is given by:


$$\begin{array}{c} i\hbar \frac{d}{dt}\Psi (t)=H(\theta )\Psi (t) \end{array}$$ (1)

As expressed in Eq. (1), the time evolution of the quantum state is governed by the parameterized Hamiltonian. Here, i denotes the imaginary unit and ℏ is the reduced Planck constant. The parameter vector θ comprises the set of physical quantities governing the interaction, including detuning, Rabi frequencies, and relative phases of the Raman fields.

Although the atom–light interaction in a Raman atom interferometer is inherently time-dependent due to pulsed laser excitation, the temporal profile of the Raman pulses is assumed to be known, deterministic, and precisely controlled. Under this assumption, the explicit time dependence of the Hamiltonian can be separated from the unknown physical parameters of interest, allowing the system to be expressed in a parameterized form. Accordingly, the Hamiltonian introduced in Eq. (1) is written as a function of a set of slowly varying or constant parameters θ, defined as:


$$\begin{array}{c} \theta =\left\{k_{\text{eff}},\delta_{\text{12}},\Delta ,\Omega_{\text{eff}},\phi_{\text{eff}}\right\} \end{array}$$ (2)

where, $k_{𝐞𝐟𝐟}$ denotes the effective Raman wave vector, $\delta_{12}$ is the two-photon detuning, Δ represents the single-photon detuning, $\Omega_{𝐞𝐟𝐟}$ is the effective Rabi frequency, and $\phi_{𝐞𝐟𝐟}$ is the relative phase of the Raman fields. This parametrization establishes a direct connection between the underlying quantum dynamics and the interferometric phase used for parameter identification via the Extended Kalman Filter (EKF).

While the complete quantum-state evolution during the Raman interaction (encompassing population transfer and momentum exchange) is well established, the present formulation leverages Eq. (1) primarily as a compact parametric model. In this paper, only the dependence of the interferometric phase on θ is required for inertial sensing and estimation purposes. For completeness, the full analytical expressions of the quantum-state evolution are provided in Appendix A in S1 Data.

#### Interferometric phase formation.

In a MZAI, the total interferometric phase shift consists of distinct contributions arising from the Raman laser phases and inertial effects acting on the atomic trajectories. These contributions can be treated independently and subsequently combined to obtain the total phase response of the interferometer.

The laser-induced phase contribution, arising from the optical phases imparted at each Raman pulse interaction, is expressed as:


$$\begin{array}{c} \Delta \Phi_{L}=\phi_{\text{1}}-\text{2}\phi_{\text{2}}+\phi_{\text{3}} \end{array}$$ (3)

where $\Delta \Phi_{L}$ denotes the laser-induced phase contribution to the total interferometric phase, and $\phi_{i}(i=\text{1},\text{2},\text{3})$ represents the optical phase of the Raman laser field evaluated at the i-th pulse interaction time. When the atomic ensemble is subjected to a constant acceleration a, the corresponding interferometric phase shift is expressed as:


$$\begin{array}{c} \Delta \Phi_{a}=k_{\text{eff}}aT^{\text{2}} \end{array}$$ (4)

where $\Delta \Phi_{a}$ denotes the acceleration-induced interferometric phase shift, $k_{\text{eff}}$ the effective Raman wave vector, a represents the constant acceleration, and T represents the interrogation time interval between successive Raman pulse interactions along the sensitive axis of the interferometer. A rotation rate Ω applied to the interferometer induces a Sagnac phase shift that scales with the effective enclosed area of the interferometer, expressed as:


$$\begin{array}{c} \Delta \Phi_{\Omega }=\text{2}{\text{A}}_{geom}\cdot \Omega \end{array}$$ (5)

here, ${\text{A}}_{geom}$ denotes the effective geometrical area enclosed by the interferometer. These phase contributions jointly determine the total interferometric phase and explicitly highlight its dependence on the effective wave vector, pulse timing, and interferometer geometry. This decomposition provides the physical foundation for the phase-based measurement model adopted in the subsequent EKF formulation.

**Fig 3:**
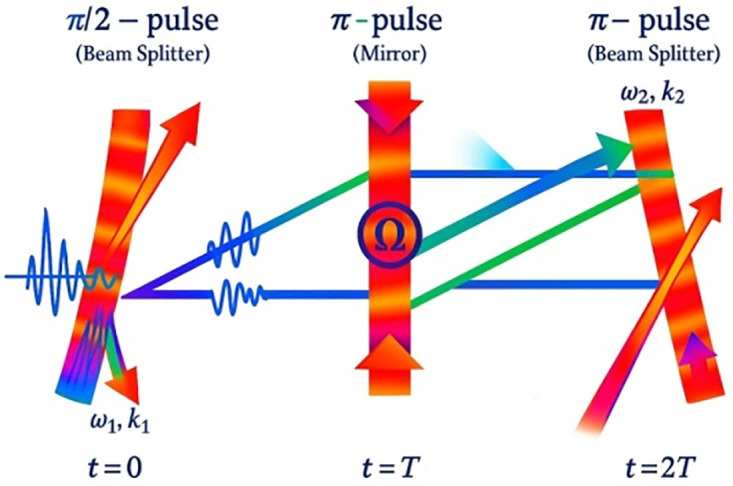
Three-pulse Mach–Zehnder Raman atom interferometer.

In the standard MZAI employing a three-pulse Raman sequence (π/2 − π − π/2), the effective geometrical area $A_{geom}$ is determined by the momentum transfer imparted by the Raman beams and by the interrogation time T between successive pulses. This configuration defines the fundamental space–time area enclosed by the two interferometric paths and therefore sets the baseline sensitivity of the interferometer to inertial effects, as illustrated in Fig 3.

**Fig 4:**
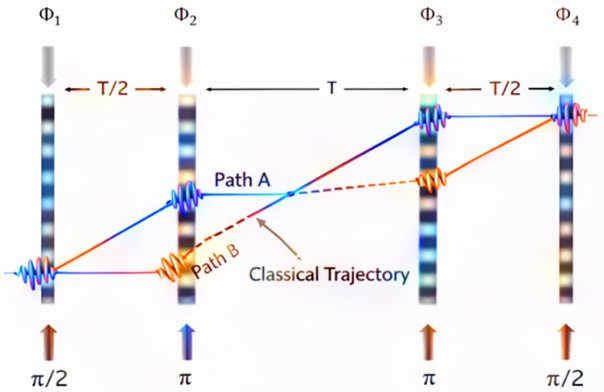
Four-pulse Raman atom interferometer with enlarged enclosed area.

To enhance inertial sensitivity, extended Raman pulse sequences such as the four-pulse configuration (π/2 − π − π − π/2) can be employed. The additional π pulse modifies the atomic trajectories by introducing an extra momentum exchange, leading to an increased separation between the interferometric paths. As a consequence, the enclosed space–time area of the interferometer is enlarged, resulting in an increased effective geometrical area $A_{geom}$, accordingly, an enhanced rotation-induced Sagnac phase response [37], This effect is schematically depicted in Fig 4.

It is important to note that the use of additional Raman pulses primarily affects the geometrical contribution to the interferometric phase, while the functional forms of the laser-induced and acceleration-induced phase terms remain unchanged. Therefore, multi-pulse interferometer geometries provide a systematic means of enhancing sensitivity to inertial quantities, particularly rotation, without altering the fundamental phase formation mechanism.

#### Phase-based measurement model for inertial sensing.

By combining the previously derived phase contributions, the total interferometric phase can be formulated as a nonlinear function of the atomic parameters and inertial quantities as follows:


$$\begin{array}{c} \Delta \Phi =f(\theta ,a,\Omega ) \end{array}$$ (6)

Since the interferometric phase encapsulates the combined effects of atomic parameters and inertial quantities, it serves as the primary physical link between the atom–light interaction dynamics and the measurable output of the interferometric sensor. In practice, the observable signal corresponds to the transition probability of atoms detected in a given hyperfine state, which exhibits a sinusoidal dependence on the interferometric phase and can be expressed as:


$$\begin{array}{c} p=A\text{cos}(\Delta \Phi +\pi /\text{2})+p_{\text{0}} \end{array}$$ (7)

where A denotes the fringe contrast (signal amplitude), and $p_{0}$ represents the mean transition probability (offset). It is important to emphasize that the geometrical area $A_{geom}$, which appears in the rotation-induced phase term, is fundamentally different from the amplitude A in Eq. (7). Specifically, $A_{geom}$ is a geometric property of the interferometer that governs the coupling between rotation and phase shift, whereas A characterizes the visibility of the interference fringes.

This phase-based observation model constitutes the nonlinear measurement equation employed in the EKF framework for parameter identification and sensor calibration, as detailed in the following section.

### Parameter estimation of EKF

Although the EKF is a well-known technique for recursive nonlinear estimation, it relies on a linear approximation of inherently nonlinear models. The underlying assumptions of the EKF stem from the way Gaussian random variables (GRVs) are propagated through the system dynamics for covariance update. In the EKF, the state distribution is approximated as a Gaussian random variable, which is then propagated analytically using a first-order linearization of the nonlinear dynamics. This approximation may introduce errors in the estimation of the true posterior mean and covariance of the transformed distribution, leading to suboptimal performance and, in some cases, filter divergence.

**Fig 5:**
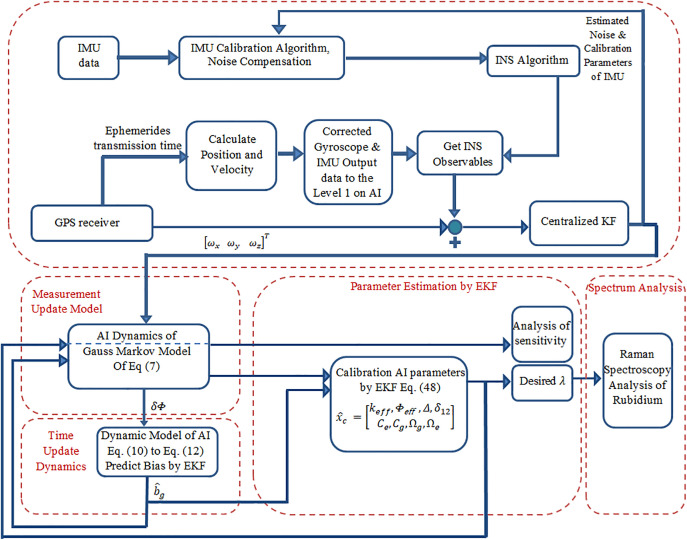
Schematic block diagram for parameter estimation process of AI Rate Integrator.

In this paper, alongside the modeling of the AI system, an experimental setup is directly employed to acquire real-world test data for the purpose of alignment and parameter estimation of the atomic interferometry sensor using GPS-aided outputs within an Extended Kalman Filter (EKF) framework. Specifically, a software-in-the-loop AI system is designed to process real rotation rate data generated by two high-grade IMUs, namely AIDIS 16407 and Vitans. The errors of these IMUs are effectively mitigated through tight integration with high-accuracy GPS measurements within an INS/GPS Kalman filter. The resulting refined rotation rate data from the AIDIS 16407 and Vitans IMUs are then incorporated into the AI rate integrator model to estimate the remaining sensor bias using the EKF. Since the measurement model of δΦ includes nonlinear dependencies on the AI sensor bias and the effective wave vector $k_{\text{eff}}$, a two-stage cascaded EKF structure is designed. As illustrated in Fig 5, the integrated INS/GPS data from the AIDIS 16407 and Vitans systems provide the reference inputs for the AI rate integrator measurement model.

Therefore, system identification of the AI sensor and estimation of its parameters through GPS-based navigation mechanization enable calibration and performance enhancement of the AI-based orientation determination system. Multiple experiments were conducted along different trajectories.

#### AI dynamics of parameters.

Before introducing the stochastic state dynamics used in the estimation framework, it is essential to clarify the role and observability characteristics of the atom interferometer (AI) parameters. Although all parameters originate from the underlying atom–light interaction model, only a subset has a direct and strongly observable influence on the interferometric phase through the measurement equation.

In particular, the effective laser phase $\Phi_{𝐞𝐟𝐟}$ and the inertial rate bias $b_{g}$ represent two fundamentally distinct sources of phase contribution in the proposed framework. The parameter $\Phi_{𝐞𝐟𝐟}$ characterizes the optical phase imprinted during Raman interactions and is treated as a calibration parameter associated with the atomic Hamiltonian. In contrast, the bias term $b_{g}$ originates from imperfections in the inertial rate integration and influences the interferometric phase indirectly through the atomic kinematic evolution.

Consequently, $b_{g}$ should not be interpreted as a surrogate for $\Phi_{𝐞𝐟𝐟}$, but rather as an additional state introduced at the measurement level to capture inertial drift effects within the EKF formulation.

Based on the phase-based observation model, the AI parameters can be classified into two categories:

1) Directly observable parameters, which have a dominant and explicit influence on the interferometric phase and can be reliably estimated from the measurement data: $k_{\text{eff}}$, $b_{g}$.
2) Auxiliary calibration parameters, which affect the interferometer response indirectly through higher-order couplings, scale factors, or contrast-related terms: $\delta_{\text{1}\text{2}}$, Δ, $C_{e},C_{g}$, $\Omega_{e},\Omega_{g}$.

This classification motivates the adoption of a cascaded estimation strategy. Parameters that explicitly appear in the interferometric phase and exhibit strong observability are estimated first with higher confidence. The remaining auxiliary parameters, which are not directly observable from the interferometric output, are then introduced as latent calibration states. Their estimation is supported by the previously identified strongly observable parameters, enabling consistent calibration and uncertainty propagation within the EKF framework.

First-order Gauss–Markov dynamics are assumed for the estimation of the remaining AI parameters, which typically describe the state evolution of the AI rate integrator. Based on the general system dynamics and the adopted hybrid EKF framework, the first-order Gauss–Markov models of the AI rate integrator parameters are expressed as:


$$\begin{array}{c} {\dot{K}}_{\text{eff}}=-\frac{\text{1}}{\tau_{\text{1}}}k_{\text{eff}}+w_{\text{1}}, \end{array}$$

$$\begin{array}{c} {\dot{\phi }}_{\text{eff}}=-\frac{\text{1}}{\tau_{\text{2}}}\phi_{\text{eff}}+w_{\text{2}}, \end{array}$$

$$\begin{array}{c} {\dot{C}}_{e}=-\frac{\text{1}}{\tau_{\text{3}}}C_{e}+w_{\text{3}},{\dot{C}}_{g}=-\frac{\text{1}}{\tau_{\text{4}}}C_{g}+w_{\text{4}}, \end{array}$$

$$\begin{array}{c} {\dot{\Omega }}_{e}=-\frac{\text{1}}{\tau_{\text{5}}}\Omega_{e}+w_{\text{5}},{\dot{\Omega }}_{g}=-\frac{\text{1}}{\tau_{\text{6}}}\Omega_{g}+w_{\text{6}}, \end{array}$$

$$\begin{array}{c} {\dot{\delta }}_{\text{12}}=-\frac{\text{1}}{\tau_{\text{7}}}\delta_{\text{12}}+w_{\text{7}}, \end{array}$$

$$\begin{array}{c} \dot{\Delta }=-\frac{\text{1}}{\tau \text{8}}\Delta +w\text{8} \end{array}$$ (8)

The correlation time of Gauss-Markov dynamics are shown by $\tau_{1}$ through $\tau_{8}$ and signals $w_{1}$ to $w_{8}$ represent white Gaussian process noises. These stochastic dynamics serve as regularization models within the EKF.

#### Measurement system model.

Although atom–light interaction is fundamentally described at the quantum-state level by the Schrödinger equation, it is important to emphasize that the EKF implemented in this work does not operate on the atomic wavefunction itself. Instead, the EKF relies on a reduced and physically interpretable representation of the measurements. In particular, the interferometric phase serves as an intermediate quantity linking the underlying quantum dynamics and atomic motion to the measurable transition probability at the interferometer output.

The estimation framework adopted in this section is formulated at the phase level rather than at the wavefunction level. The parametric Schrödinger model is used solely to establish the physical origin and parameter dependence of the interferometric phase, while the EKF measurement model directly exploits phase-dependent observable quantities.

In literature, e.g., [38,39], the atom phase shift caused by inertial force of a turn rate is often expressed directly in relationship to the inertial Coriolis acceleration by turn rate. This mostly makes sense in scenarios with a nearly constant value like in gravimetry measurements. However, involving more general advantages of rate integrator sensor and its turning to accelerometer through in connection with Coriolis force, the measured phase output is correspondent to the atomic variables, x as:


$$\begin{array}{c} \Phi =kx \end{array}$$ (9)

Eq. (9) provides a concise representation of the phase imprinting experienced by the atomic wavepacket during a Raman interaction. The accumulated phase is directly proportional to the atom’s position within the laser field, with the effective laser wave vectorkx~1/λ defined by the laser wavelength. This local and physically interpretable relationship serves as the foundational link between the atomic motion and the measurable interferometric phase. By establishing this connection, Eq. (9) enables the construction of a phase-level measurement model, which is exploited by the EKF framework to infer atomic and inertial parameters without requiring full quantum-state propagation.

In remainder of the research paper, the phase shift between the two atom paths is deﬁned by each of the interactions of the atom clouds with the interrogation lasers. The Mach-Zehnder conﬁguration, uses a series of three laser pulses (π/2 − π − π/2) to split, reverse and recombine the two atom paths, as their internal states. The just described algorithm works as long as the difference of the AI phase shift and the predicted phase shift of the conventional IMU, including its errors like bias, is less than π/2. Based on this phase-position relationship, the EKF state vector is constructed to capture the kinematic quantities that directly influence phase accumulation during the interrogation sequence. Hence, the atomic state vector is constructed as:


$$\begin{array}{c} x_{s}={\left[q^{b},v^{b},b_{g}\right]}^{T} \end{array}$$ (10)

here, $q^{b}$, $v^{b}$ represents the atom position and velocity in the body frame along the track direction affected by the Coriolis force/acceleration of the input rotation rate $\Omega^{b}$ and the corresponding bias, $b_{g}$ of AI rate sensor. This reduced yet physically meaningful representation captures all relevant information needed to model the interferometric phase within the EKF framework. The kinematical evolution of the atomic position and velocity, coupled with the AI bias, is then described by the following dynamical system:


$$\begin{array}{c} {\dot{q}}^{b}=v^{b} \end{array}$$ (11)

$$\begin{array}{c} {\dot{v}}^{b}={\text{2}\Omega }^{b}*v^{b}+{\text{2}b}_{g}*v^{b} \end{array}$$ (12)

$$\begin{array}{c} {\dot{b}}_{g}=\text{0} \end{array}$$ (13)

Using the state dynamics defined in Eqs. (11)–(13), the atomic trajectories can be propagated forward in time to obtain the predicted motion of the interferometric system. These predicted states are then mapped into the measurement domain through the interferometric phase relation. Accordingly, the measurement model for the EKF is formulated based on the phase evaluation at the Raman pulse interaction times, leading to the following expression:


$$\begin{array}{c} \Phi =k\text{eff }\left(q^{b}\left(Tf\right)-\text{2}q^{b}\left(\frac{\text{1}}{\text{2}}Tf\right)\right) \end{array}$$ (14)

where $q^{b}\left(T_{f}\right)$ represents the atomic position in the body frame at the final interrogation time $T_{f}$, and $q^{b}\left(\frac{\text{1}}{\text{2}}T_{f}\right)$ corresponds to the intermediate position at half of the interrogation sequence.

The actual measurement provided by the atom interferometer is the transition probability between the hyperfine states. This probability follows a sinusoidal pattern determined by the interferometric phase, δΦ and the relevant state parameters. It therefore constitutes the observation input for the EKF, linking the physical phase evolution to the filter update.


$$\begin{array}{c} p=A\text{cos}\left(\Phi_{L}+\Phi_{IMU}+\delta \Phi \right)+p_{\text{0}} \end{array}$$ (15)

The amplitude A and the DC offset $p_{0}$ are assumed to be known constants. The laser phase $\Phi_{L}$ is regulated by the controller such that the sum $\Phi_{L}+\Phi_{IMU}$ is maintained at the operating point π/2. The error phase shift δΦ is the part that results in from any errors of the accelerations measured by the conventional IMU, here the biases. It couples the observation equation with the state parameters.

This phase error couples the physical state parameters to the measurement, providing a direct link between the system dynamics and the EKF observation. During each measurement cycle, the integrated rate bias produces a velocity increment that manifests as the observed δΦ, thereby connecting the phase-based measurement to the underlying atomic motion.


$$\begin{array}{c} \delta v=b_{g}T_{f} \end{array}$$ (16)

substituting Eq. (16) into the phase relation yields the corresponding expression for the interferometric phase shift:


$$\begin{array}{c} \delta \Phi =\frac{\text{1}}{\text{4}}k_{\text{eff}}b_{g}\overset{\text{2}}{\underset{f}{T}} \end{array}$$ (17)

incorporation of the accumulated phase error into the observation equation leads to a fully phase-based nonlinear measurement model suitable for EKF implementation.


$$\begin{array}{c} p=Acos\left(\frac{\pi }{\text{2}}+\frac{\text{1}}{\text{4}}k_{\text{eff}}b_{g}\overset{\text{2}}{\underset{f}{T}}\right)+p_{\text{0}}=-Asin\left(\frac{\text{1}}{\text{4}}k_{\text{eff}}b_{g}\overset{\text{2}}{\underset{f}{T}}\right)+p_{\text{0}} \end{array}$$ (18)

Eqs. (15)–(18) clearly show that the EKF measurement framework operates entirely at the phase level. Rather than estimating full atomic states or wavefunctions, the filter exploits the nonlinear dependence of the observable transition probability on the interferometric phase. The accumulated phase error δΦ serves as the coupling term linking the physical parameters of the atom interferometer to the measurable output. Substituting this phase error into the observation equation produces a fully phase-based, nonlinear measurement model, which explicitly depends on the effective Raman wave vector $k_{\text{eff}}$ and the AI rate bias $b_{g}$) these quantities constitute the dominant(, directly observable states within the proposed EKF framework.

### Hybrid EKF Framework with Continuous-Time AI Dynamics and Discrete-Time Measurements

Although the atomic dynamics governing the evolution of the interferometer states are naturally described in continuous time, the atom interferometer provides measurements only at discrete instants corresponding to the completion of each interrogation cycle. Consequently, the estimation problem considered in this work has an inherently hybrid structure, in which a continuous-time dynamical model is combined with discrete-time measurement updates.

In the proposed framework, the a priori propagation of the state estimate and its associated error covariance is driven by the continuous-time system dynamics derived in Eqs. (8) and (11)–(13), which describe the kinematic evolution of atomic position, velocity, and bias under inertial effects. These equations capture the physical motion of the atomic wave packet and constitute the process model of the EKF.

In contrast, the measurements are available only at discrete instants, corresponding to the transition probability output of the atom interferometer at the end of each interrogation cycle. These observations are incorporated through a nonlinear measurement model, leading to an a posteriori correction of both the state estimate and its covariance. This structure naturally results in a hybrid EKF formulation, combining continuous-time state propagation with discrete-time measurement updates.

For the proposed hybrid EKF, the system dynamics are modeled in continuous time as $\dot{x}=f(x)$, while the measurements are incorporated in discrete time through the measurement model $z_{k}=h(x_{k})$, as expressed by:


$$\begin{array}{c} \dot{x}=f(x)+w, \end{array}$$

$$\begin{array}{c} z_{k}=h\left(x_{k}\right)+v_{k} \end{array}$$ (19)

The process noise w and the measurement noise $v_{k}$ are modeled as zero-mean Gaussian sequences with covariance matrices 𝐐 and ${𝐑}_{𝐤}$, respectively. The hybrid EKF is initialized with the a posteriori state estimate $\overset{+}{\underset{s_{0}}{\hat{x}}}=x_{0}=0_{3*3}$ and the corresponding error covariance matrix $\overset{+}{\underset{s_{0}}{P}}=P_{0}$. In this work, the initial state is assumed to be zero-mean, reflecting the absence of prior knowledge about the atom displacement, velocity, and bias states. Based on the continuous-time system dynamics introduced in Eq. (10), the time-update (prediction) step of the EKF propagates both the state estimate and its covariance over each sampling interval from k−1 to k. The predicted state and covariance are obtained by integrating the nonlinear state equation and the associated Riccati equation as:


$$\begin{array}{c} \overset{-}{\underset{s_{k}}{\dot{\hat{x}}}}=f\left(\overset{-}{\underset{s_{k}}{\hat{x}}}\right),K=\text{1},\text{2},\ldots \end{array}$$

$$\begin{array}{c} \overset{-}{\underset{s_{k}}{\hat{x}}}=\overset{+}{\underset{s_{k-\text{1}}}{\hat{x}}}, \end{array}$$

$$\begin{array}{c} \overset{-}{\underset{s_{k}}{\dot{P}}}=A_{s}\overset{-}{\underset{s_{k}}{P}}+\overset{-}{\underset{s_{k}}{P}}{A_{s}}^{T}+Q_{s}, \end{array}$$

$$\begin{array}{c} \overset{-}{\underset{s_{k}}{P}}=\overset{+}{\underset{s_{k-\text{1}}}{P}}, \end{array}$$

$$\begin{array}{c} As=\frac{\partial fs}{\partial xs}|x=\overset{+}{\underset{sk-\text{1}}{\hat{x}}} \end{array}$$ (20)

where $A_{s}$ denotes the Jacobian matrix of the system dynamics with respect to the state vector, evaluated at the previous a posteriori estimate. The predicted state estimate $\overset{-}{\underset{s_{k}}{\hat{x}}}$ and its associated covariance $\overset{-}{\underset{s_{k}}{P}}$, obtained from the continuous-time time-update equations in Eqs. (19)–(20), are then used at each measurement instant to perform the discrete-time measurement update. Specifically, these predicted quantities are employed to compute the Kalman gain $K_{s_{k}}$, followed by the state update $\overset{+}{\underset{s_{k}}{\hat{x}}}$ and the covariance update $\overset{+}{\underset{s_{k}}{P}}$, as given by:


$$\begin{array}{c} K_{s_{k}}=\overset{-}{\underset{s_{k}}{P}}\overset{T}{\underset{s_{k}}{H}}{\left(H_{s_{k}}\overset{-}{\underset{s_{k}}{P}}\overset{T}{\underset{s_{k}}{H}}+R_{s_{k}}\right)}^{-\text{1}}, \end{array}$$

$$\begin{array}{c} \overset{+}{\underset{s_{k}}{\hat{x}}}=\overset{-}{\underset{s_{k}}{\hat{x}}}+K_{s_{k}}\left[z_{s_{k}}-h_{s_{k}}\left(\overset{-}{\underset{s_{k}}{\hat{x}}},t_{k}\right)\right],t_{k}=kt \end{array}$$

$$\begin{array}{c} \overset{+}{\underset{s_{k}}{P}}=\left(I-K_{s_{k}}H_{s_{k}}\right)\overset{-}{\underset{s_{k}}{P}}{\left(I-K_{s_{k}}H_{s_{k}}\right)}^{T}+H_{s_{k}}\overset{-}{\underset{s_{k}}{P}}\overset{T}{\underset{s_{k}}{H}} \end{array}$$ (21)

Eq. (21) define the standard discrete-time measurement update of the EKF, including the computation of the Kalman gain, the state correction, and the covariance update. Since the phase-based measurement model in Eqs. (17) and (18) depends explicitly on both the AI rate bias $b_{g}$ and the effective wave vector $k_{\text{eff}}$, a single EKF is generally insufficient to ensure reliable and well-conditioned estimation of all parameters. To address this issue, a cascade EKF architecture is adopted. In the proposed cascade structure, the first EKF focuses on estimating the atomic kinematic states and the AI rate bias using the phase-derived measurement. The estimated bias is subsequently provided as an input to a second EKF, which is dedicated to the identification of intrinsic AI parameters and laser-related coefficients. For the first EKF, the system dynamics in Eqs. (11)–(13) define the state vector $x_{s}=[x_{s_{\text{1}}},x_{s_{\text{2}}},x_{s_{\text{3}}}]$, corresponding to atomic position, velocity, and AI rate bias, respectively. Based on the phase-based observation model in Eq. (18), the measurement equation of the first EKF can be explicitly written as:


$$\begin{array}{c} z_{s_{k}}=z_{k}\left(x_{s}\right)=p=-\frac{\text{1}}{\text{4}}A{\hat{k}}_{\text{eff}}\overset{\text{2}}{\underset{f}{T}}b_{g}+v_{s}=-\frac{\text{1}}{\text{4}}A{\hat{k}}_{\text{eff}}\overset{\text{2}}{\underset{f}{T}}x_{s_{{\text{3}}_{k}}}+v_{s} \end{array}$$

$$\begin{array}{c} H_{s_{k}}={\frac{\partial h\left(x_{s}\right)}{\partial x_{s}}|}_{x_{s}=\overset{-}{\underset{s_{k}}{\hat{x}}}}=\left[\text{0}\text{0}-\text{0}.\text{2}\text{5}A{\hat{k}}_{\text{eff}}\overset{\text{2}}{\underset{f}{T}}\right] \end{array}$$ (22)

where $v_{s}$ denotes the measurement noise associated with the transition probability. The measurement noise covariance is defined as $R_{s}={\text{10}}^{-\text{5}}$, as used in the EKF update in Eq. (21). The process noise vector is denoted by w, which represents the stochastic disturbances affecting the system dynamics. Accordingly, the process noise covariance matrix and the initial state covariance matrix are selected as:


$$\begin{array}{c} Q_{s}=cov(w)=diag\left[{\text{10}}^{-\text{10}}{,\text{ 10}}^{-\text{5}},{\text{10}}^{-\text{5}}\right], \end{array}$$

$$\begin{array}{c} P_{\text{0}}=diag\left[{\text{10}}^{\text{5}}{,\text{ 10}}^{\text{5}},{\text{10}}^{\text{6}}\right] \end{array}$$ (23)

where a diagonal matrix is constructed from the given elements. These values reflect the relative confidence in the initial atomic kinematic states and the slow temporal variation of the AI rate bias.

The continuous-time dynamics of the first EKF are governed by Eqs. (11)–(13). Embedding these dynamics into the EKF time-update yields the state propagation equation, while the associated Jacobian matrix $A_{s}$, required for covariance propagation in Eq. (20), is obtained by linearizing the nonlinear system dynamics with respect to the state vector $x_{s}$ around the predicted state estimate.


$$\begin{array}{c} \overset{-}{\underset{s}{\dot{\hat{x}}}}=f\left(\overset{-}{\underset{s}{\hat{x}}}\right)=f\left(\overset{+}{\underset{s_{k-\text{1}}}{\hat{x}}}\right)=[\begin{array}{c} \overset{+}{\underset{{\text{s}}_{{\text{2}}_{\text{k}-\text{1}}}}{\hat{\text{x}}}}\\ {\text{2}\Omega }^{\text{b}}\overset{+}{\underset{{\text{s}}_{{\text{2}}_{\text{k}-\text{1}}}}{\hat{\text{x}}}}+\text{2}\overset{+}{\underset{{\text{s}}_{{\text{2}}_{\text{k}-\text{1}}}}{\hat{\text{x}}}}\overset{+}{\underset{{\text{s}}_{{\text{3}}_{\text{k}-\text{1}}}}{\hat{\text{x}}}}\\ \text{0} \end{array}], \end{array}$$

$$\begin{array}{c} A_{s}={\frac{\partial f\left(x_{s}\right)}{\partial x_{s}}|}_{x_{s}=\overset{+}{\underset{s_{k-\text{1}}}{\hat{x}}}}=[\begin{array}{ccc} \text{0} & \text{1} & \text{0}\\ \text{0} & {\text{2}\Omega }^{b}+\text{2}\overset{+}{\underset{s_{{\text{3}}_{k-\text{1}}}}{\hat{x}}} & \text{2}\overset{+}{\underset{s_{{\text{2}}_{k-\text{1}}}}{\hat{x}}}\\ \text{0} & \text{0} & \text{0} \end{array}] \end{array}$$ (24)

by applying the EKF formulation given in Eqs. (20)–(24), the atomic displacement, velocity, and the AI rate bias are estimated. These kinematic states constitute the state vector $x_{s}$ and provide the required bias information for parameter identification. Based on the estimated bias obtained from the first EKF, a second EKF is constructed to estimate the intrinsic parameters of the atom interferometer. This hybrid EKF employs the continuous-time parameter dynamics of Eq. (8) together with the discrete-time phase-related measurements described in Eqs. (14)–(18). In this framework, the measurement update equations are reformulated as a nonlinear regression with respect to the parameter vector $x_{c}$.


$$\begin{array}{c} x_{c}={\left[k_{\text{eff }},\Phi_{\text{eff }},\Delta {,\delta }_{\text{12}},C_{e},C_{g},\Omega_{g},\Omega_{e}\right]}^{T} \end{array}$$ (25)

Based on Eq. (17), the measurement model can be formulated as a nonlinear regression function of the atom interferometer parameter vector $x_{c}$, leading to the following expression:


$$\begin{array}{c} \delta \Phi =\frac{\text{1}}{\text{4}}{\hat{b}}_{g}\overset{\text{2}}{\underset{f}{T}}k_{\text{eff}}, \end{array}$$

$$\begin{array}{c} \omega_{eg}-\frac{p^{\text{2}}}{\text{2}m\text{ħ}}=\delta_{\text{12}}-\Delta -\left(\frac{{\text{ħ}}^{\text{2}}p^{\text{2}}}{\text{2}m}k_{\text{eff}}\right), \end{array}$$

$$\begin{array}{c} sin\left(\frac{{(\Omega }_{e}-\Omega_{g})\tau }{\text{2}}\right)=\frac{C_{g}}{C_{e}}, \end{array}$$

$$\begin{array}{c} \delta \Phi -\omega_{\text{1}}t{=k_{\text{eff}}{\hat{b}}_{g}t^{\text{2}}-cos}^{-\text{1}}\left(\frac{\Omega_{g}}{E_{\text{1}}}\right), \end{array}$$

$$\begin{array}{c} \delta \Phi -\omega_{\text{2}}t{=k_{\text{eff}}{\hat{b}}_{g}t^{\text{2}}-cos}^{-\text{1}}\left(\frac{\Omega_{e}}{E_{\text{2}}}\right) \end{array}$$ (26)

The parameter estimation EKF is implemented according to Eq. (20), while accounting for the parameter dynamics defined in Eq. (8). Accordingly, the time-update equations and the corresponding state transition matrices are derived as follows:


$$\begin{array}{c} \overset{-}{\underset{c_{k}}{\dot{\hat{x}}}}=f\left(\overset{-}{\underset{c_{k}}{\hat{x}}}\right),k=\text{1},\text{2},\ldots \end{array}$$

$$\begin{array}{c} \overset{-}{\underset{c_{k}}{\hat{x}}}=\overset{+}{\underset{c_{k-\text{1}}}{\hat{x}}}, \end{array}$$

$$\begin{array}{c} \overset{-}{\underset{c_{k}}{\dot{P}}}=A_{c}\overset{-}{\underset{c_{k}}{P}}+\overset{-}{\underset{c_{k}}{P}}{A_{c}}^{T}+Q_{c}, \end{array}$$

$$\begin{array}{c} \overset{-}{\underset{c_{k}}{P}}=\overset{+}{\underset{c_{k-\text{1}}}{P}} \end{array}$$ (27)

Based on the parameter definition in Eq. (25), the continuous-time parameter dynamics are modeled as follows:


$$\begin{array}{c} f\left(x_{c}\right)=col\left[-\frac{\text{1}}{\tau_{i}}{x_{c}}_{i}\right], \end{array}$$

$$\begin{array}{c} A_{c}=diag\left[-\frac{\text{1}}{\tau_{i}}\right],\;i=\text{1},\ldots ,\text{8} \end{array}$$ (28)

where col and diag denote the column vector and diagonal matrix operators, respectively. Based on the time-update results $\overset{-}{\underset{c}{\hat{x}}}$ and $\overset{-}{\underset{c_{k}}{P}}$ obtained from Eq. (27), the EKF measurement update given in Eq. (21) is then applied for parameter estimation using the corresponding observation vector and covariance matrices.


$$\begin{array}{cc} K_{c_{k}} & =\overset{-}{\underset{c_{k}}{P}}\overset{T}{\underset{c_{k}}{H}}{\left(H_{c_{k}}\overset{-}{\underset{c_{k}}{P}}\overset{T}{\underset{c_{k}}{H}}+R_{c_{k}}\right)}^{-\text{1}},\\ \overset{+}{\underset{c_{k}}{\hat{x}}} & =\overset{-}{\underset{c_{k}}{\hat{x}}}+K_{c_{k}}\left[z_{c_{k}}-h_{c_{k}}\left(\overset{-}{\underset{c_{k}}{\hat{x}}},t_{k}\right)\right],t_{k}=kt\\ \overset{+}{\underset{c_{k}}{P}} & =\left(I-K_{c_{k}}H_{c_{k}}\right)\overset{-}{\underset{c_{k}}{P}}{\left(I-K_{c_{k}}H_{c_{k}}\right)}^{T}+H_{c_{k}}\overset{-}{\underset{c_{k}}{P}}\overset{T}{\underset{c_{k}}{H}} \end{array}$$ (29)

Following the measurement model defined in Eq. (26), the corresponding observation process is formulated in a nonlinear state–space form suitable for EKF implementation, where the measurement vector is expressed as:


$$\begin{array}{c} z\left(x_{c}\right)=[\begin{array}{c} \begin{array}{c} \begin{array}{c} \delta \Phi \\ \omega_{eg}-\frac{p^{\text{2}}}{\text{2}m\text{ħ}} \end{array}\\ sin\left(\frac{{(\Omega }_{e}-\Omega_{g})\tau }{\text{2}}\right) \end{array}\\ \begin{array}{c} \delta \Phi -\omega_{\text{1}}t\\ \delta \Phi -\omega_{\text{2}}t \end{array} \end{array}]=h\left(x_{c}\right)+v_{c}, \end{array}$$

$$\begin{array}{c} H={\frac{\partial h\left(x_{c}\right)}{\partial x_{c}}|}_{x_{c}={\hat{x}}_{c}}=[\begin{array}{cccccccc} \text{0}.\text{2}\text{5}k_{\text{eff}}{\hat{b}}_{g}t^{\text{2}} & \text{0} & \text{0} & \text{0} & \text{0} & \text{0} & \text{0} & \text{0}\\ -\text{0}.\text{5}m^{-\text{1}}{\text{ħ}}^{\text{2}}p^{\text{2}} & \text{0} & -\text{1} & \text{1} & \text{0} & \text{0} & \text{0} & \text{0}\\ \text{0} & \text{0} & \text{0} & \text{0} & \text{0} & -C_{g}{C_{e}}^{-\text{2}} & {C_{e}}^{-\text{1}} & \text{0}\\ {\hat{b}}_{g}t^{\text{2}} & \text{0} & \text{0} & \text{0} & \text{0} & \text{0} & -{E_{\text{1}}\left(\text{1}+{\Omega_{g}}^{\text{2}}{E_{\text{1}}}^{-\text{2}}\right)}^{-\text{1}} & \text{0}\\ {\hat{b}}_{g}t^{\text{2}} & \text{0} & \text{0} & \text{0} & \text{0} & \text{0} & \text{0} & {{-E}_{\text{2}}\left(\text{1}+{\Omega_{e}}^{\text{2}}{E_{\text{2}}}^{-\text{2}}\right)}^{-\text{1}} \end{array}] \end{array}$$ (30)

The nonlinear measurement function in Eq. (30) is linearized around the current parameter estimate ${\hat{x}}_{c}$ to obtain the corresponding observation matrix H. In addition, ${𝐐}_{𝐜}$ denotes the covariance matrix of the process noise associated with the parameter dynamics in Eq. (6), while the residual modeling uncertainties $w={[w_{1}\ldots w_{8}]}^{T}$ are represented by the process noise vector.


$$\begin{array}{c} {\text{Q}}_{\text{C}}=\text{diag}\left[{\text{10}}^{-\text{6}}{\text{10}}^{-\text{10}}{\text{10}}^{-\text{5}}{\text{10}}^{-\text{6}}{\text{10}}^{-\text{10}}{\text{10}}^{-\text{10}}{\text{10}}^{-\text{10}}{\text{10}}^{-\text{10}}\right] \end{array}$$ (31)

In accordance with the measurement noise level $v_{c}$ defined in Eq. (26), the measurement noise covariance matrix is given by:

$R_{c}=diag\left[{\text{10}}^{-\text{10}}{\text{10}}^{-\text{10}}{\text{10}}^{-\text{5}}{\text{10}}^{-\text{6}}{\text{10}}^{-\text{10}}\right]$,


$$\begin{array}{c} P_{\text{0}}=diag\left[{\text{10}}^{\text{5}}{,\text{ 10}}^{\text{5}},{\text{10}}^{\text{6}},{\text{10}}^{\text{6}},{\text{10}}^{\text{6}},{\text{10}}^{\text{5}},{\text{10}}^{\text{5}},{\text{10}}^{\text{5}}\right] \end{array}$$ (32)

The proposed hybrid cascade EKF therefore combines continuous-time parameter dynamics with discrete-time phase-based measurements, enabling simultaneous estimation of atomic kinematics, inertial bias, and intrinsic atom interferometer parameters in a unified probabilistic framework.

## Results and discussion

### Experimental Results

In this section, we review the calibration procedure, parameter estimation, software output logging, and analysis of the results.

First, the measurement behavior is evaluated using data collected from test routes driven by a vehicle. The experiments were conducted under controlled conditions, where predefined routes and dynamic maneuvers were designed to comprehensively assess the performance of the EKF. The estimated attitude and alignment results obtained from the orientation system are consistent with those produced by the atomic interferometry rate sensor under EKF-based parameter estimation.

Two independent orientation systems are employed for data collection along the test routes. The first system consists of a low-cost magnetic IMU combined with a commercial global positioning system (GPS). The second system includes a Vitans micro-electromechanical IMU integrated with GPS and is used as a reference for validating the estimator. In both systems, gyroscope data are used as known inputs to the navigation and alignment model, while accelerometer and GPS data are used as measurement observations.

**Fig 6:**
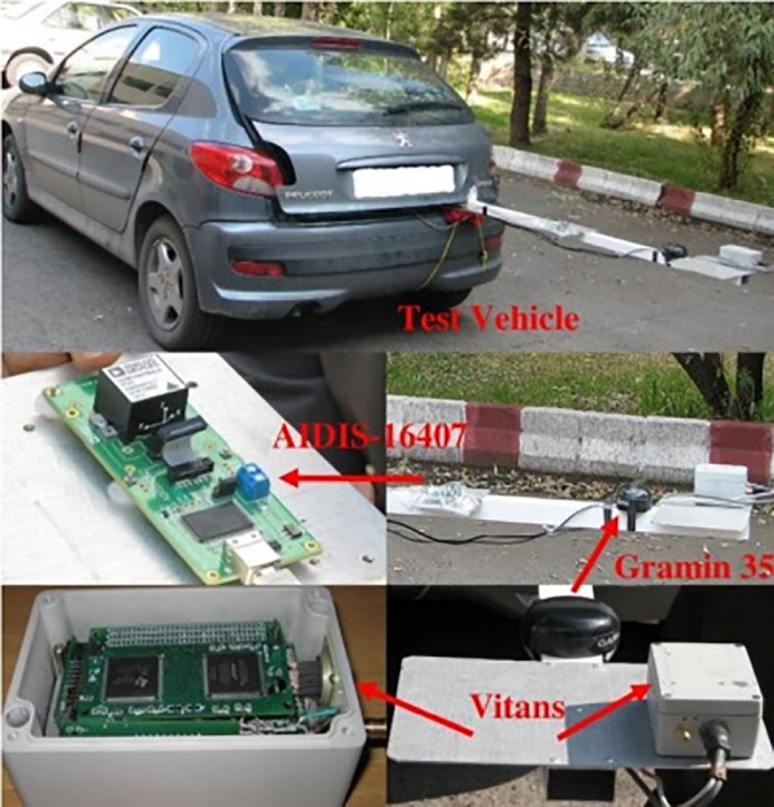
Interior view of the micro-electromechanical tests.

The interior views of the Vitans micro-electromechanical system integrated with GPS and the AIDIS16407 micro-electromechanical IMU are shown in Fig 6. The metal body of the vehicle and surrounding magnetic fields distort the Earth’s magnetic field vector, resulting in a significant perturbation in the output of the magnetic sensors. To mitigate this disturbance, an aluminum frame is designed to position the system at a sufficient distance from the vehicle body.

As shown in Fig 6, the Vitans MEMS system, GPS receiver, and AIDIS16407 IMU are rigidly mounted on an aluminum profile, ensuring negligible relative motion among them. This profile is also rigidly attached to the vehicle chassis, eliminating any relative displacement with respect to the vehicle.

The Vitans inertial system comprises orthogonal tri-axial accelerometers, gyroscopes, and magnetometers, along with pressure, temperature, and inertial sensors. An analog-to-digital converter is used to convert the sensor outputs into digital signals. Data fusion and navigation computations are performed on a high-frequency microprocessor. In this unit, the processing is carried out based on the Vitans inertial navigation algorithm, which employs quaternion algebra, time-series analysis, and optimal filtering techniques.

The vehicle followed a predefined closed-loop trajectory with varying curvature and slope to evaluate the dynamic performance of the proposed EKF-based calibration framework.

The primary objective of the experiments is to determine the reference trajectory and improve localization accuracy using GPS measurements. The identification of the gyroscope and the estimation of its parameters contribute to enhanced positioning accuracy through the EKF-based estimator.

Multiple experiments were conducted using precise position and angular measurements obtained from the Vitans and AIDIS16407 systems. These measurements were then compared with the outputs of the atomic interferometry sensor model under EKF-based parameter estimation.

Through the estimation of AI sensor parameters, the optimal wavelength was ultimately determined, resulting in improved Raman spectroscopic performance and increased sensor sensitivity.

### Estimation of AI sensor parameters

The process of identifying the relationships of the atomic interferometer and its modelling parameters through estimation results of the EKF are evaluated in comparison with the state of real true values [40]. Considering uncertainty on the measurement Eq. (24) and low noises of the practical AI sensors, it is worthy of noting that the noise covariance matrix is designed about $R_{k}=10^{-10}$. Along with applying these perfect noise specifications on the measurement signals for parameter estimation of the AI gyroscope, the process noise *w* covariance components set on 10^−6^ scale.

**Fig 7:**
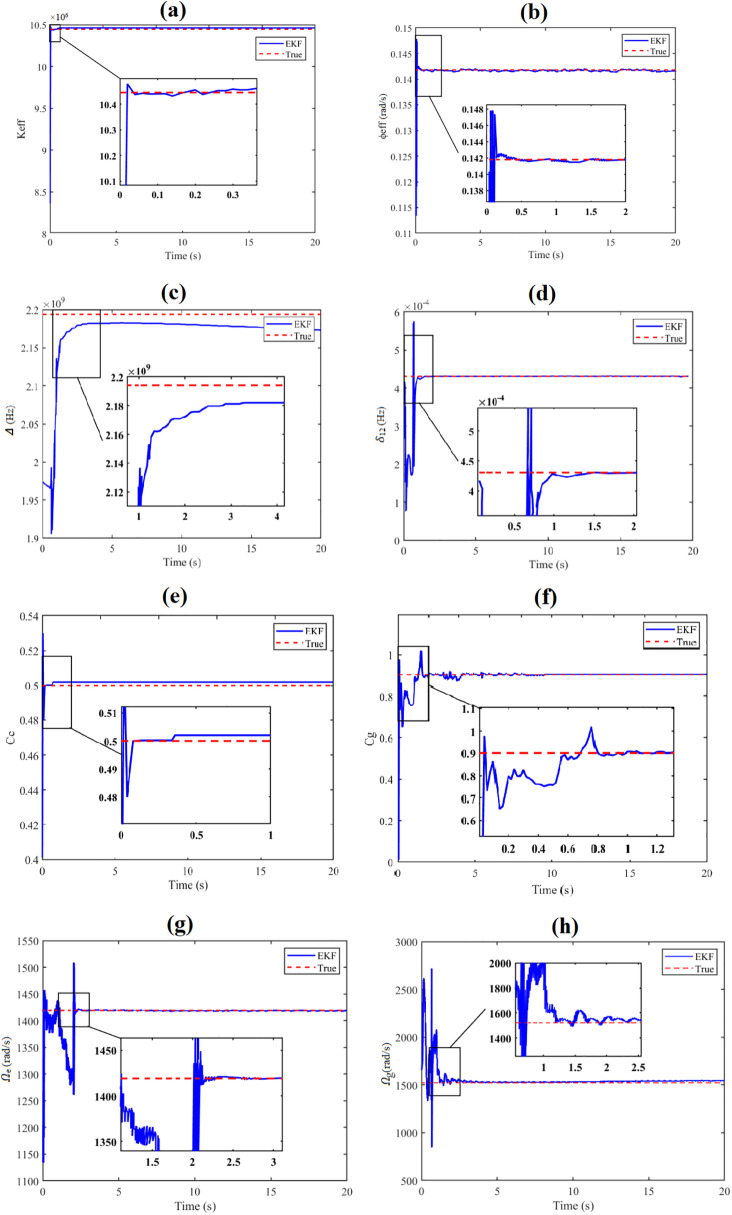
True reference value and estimated value by EKF estimator for the relevant parameters of Raman transition.

According to the assumptions and Fig 5 block-diagram, the estimation results obtained with a time constant of τ=3 sec, indicate that the EKF is capable of accurately and stably identifying the main and calibration parameters of the atomic rate integrator. Accordingly, Fig 7 presents the EKF-based estimation results of the principal and calibration parameters of the atomic rate integrator. Subfigures (a) to (h) correspond, respectively, to the estimation of $k_{𝐞𝐟𝐟},\Phi_{𝐞𝐟𝐟},\Delta ,\delta_{12},C_{e},C_{g},\Omega_{g},\Omega_{e}$, where the EKF estimates are compared with the corresponding reference true values. The results demonstrate fast convergence and well-damped transient behavior for all estimated parameters. After the initial transient phase, the residual oscillations around the reference values remain minimal [40]. This behavior reflects appropriate tuning of the process and measurement noise covariances, as well as good consistency between the dynamic model and the physical structure of the atomic sensor.

As observed in subfigures (a) and (b), the parameters $k_{𝐞𝐟𝐟},\Phi_{𝐞𝐟𝐟}$ converge rapidly and reach their true values in less than approximately 0.5 sec. In addition to fast convergence, the post-convergence estimates remain highly stable, indicating strong observability of these parameters within the phase-based measurement model. This behavior highlights the ability of the EKF to reliably identify key phase-related parameters that directly affect interferometer performance.

Subfigures (c) and (d) show the estimation results for the spectral parameters Δ and $\delta_{12}$. The results indicate that $\delta_{12}$ converges in less than approximately 1.5 s, while Δ reaches its reference value within about 3 s. Although the convergence time of these parameters is longer than that of the phase and contrast parameters, their estimation trajectories remain smooth and stable. This demonstrates that the EKF effectively manages parameter coupling and indirect sensitivity within the measurement equation.

Subfigures (e) and (f) correspond to the estimation of the contrast coefficients $C_{e}$ and $C_{g}$, which converge in less than approximately 1 s and 0.5 s, respectively. The low variance of these estimates confirms the adequacy of the sinusoidal transition probability model and indicates a favorable signal-to-noise ratio in the interferometer output.

Subfigures (g) and (h) present the estimation results for the parameters $\Omega_{g}$ and $\Omega_{e}$. These parameters converge within approximately 3.5 s and 2.5 s, respectively. Despite their slower dynamics and more indirect influence on the interferometric phase, the results show that the proposed cascade EKF structure can successfully identify these parameters with stable convergence.

The results in Fig 7 show that the proposed EKF, based on a phase-driven measurement model and properly tuned noise statistics, achieves robust and accurate simultaneous estimation of the atomic rate integrator parameters, converging within a short time.

Following estimation of the essential parameters of AI rate integrator, we obtained the desired value of λ. This main parameter is identified as 1100 *nm* which is used as input setup in the Raman spectroscopy for clearly scattering analysis of deferent elements.

### Sensitivity of Atomic Interferometer Rate Sensor under Rotation and Acceleration

To quantitatively assess the sensitivity enhancement of the atomic interferometer as a positioning and rate-sensing device, a comparative analysis between the 3-pulse and 4-pulse MZAI configurations, is performed. The evaluation is conducted by examining the effect of the estimated effective wave vector $k_{𝐞𝐟𝐟}$ on the interferometric response.

**Fig 8:**
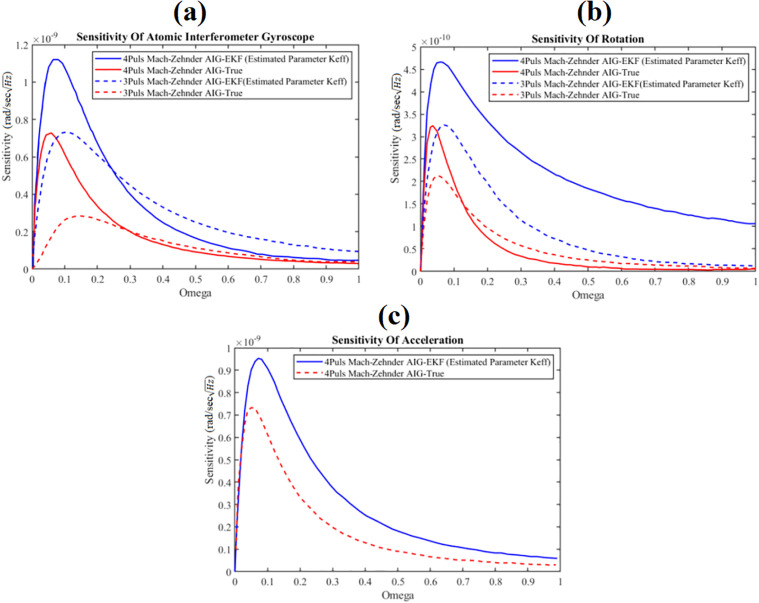
Comparison of the sensitivity of AI rate sensor.

Fig 8 (a) illustrates the sensitivity improvement achieved in the 4-pulse MZAI configuration by incorporating the EKF-estimated value of $k_{𝐞𝐟𝐟}$. The results indicate that the 4-pulse configuration attains a peak sensitivity of 11.2 × 10^−10^, compared with 7.3 × 10^−10^ obtained using the nominal (actual) value of $k_{𝐞𝐟𝐟}$. This enhancement reflects the increased phase accumulation enabled by the extended pulse sequence and the accurate calibration of the effective Raman wave vector.

The corresponding sensitivity to rotation is presented in Fig 8(b). As shown, the 4-pulse MZAI exhibits a maximum rotational sensitivity of 4.7 × 10^−10^, whereas the reference sensitivity associated with the nominal $k_{𝐞𝐟𝐟}$ is limited to 3.3 × 10^−10^. This improvement confirms that the butterfly-type interferometer geometry, combined with EKF-based parameter optimization, significantly strengthens the coupling between the Sagnac phase and the rotation rate.

Similarly, Fig 8(c) reports the acceleration sensitivity obtained for the 4-pulse MZAI configuration. The optimized system reaches a maximum sensitivity of 0.95 × 10^−10^, in contrast to 0.73 × 10^−10^when the nominal $k_{𝐞𝐟𝐟}$ is used. The observed increase demonstrates that accurate identification of interferometer parameters directly translates into enhanced inertial sensitivity, particularly for acceleration measurements that depend quadratically on the interrogation time.

### Investigating the effects on angular and orientation quality

**Fig 9:**
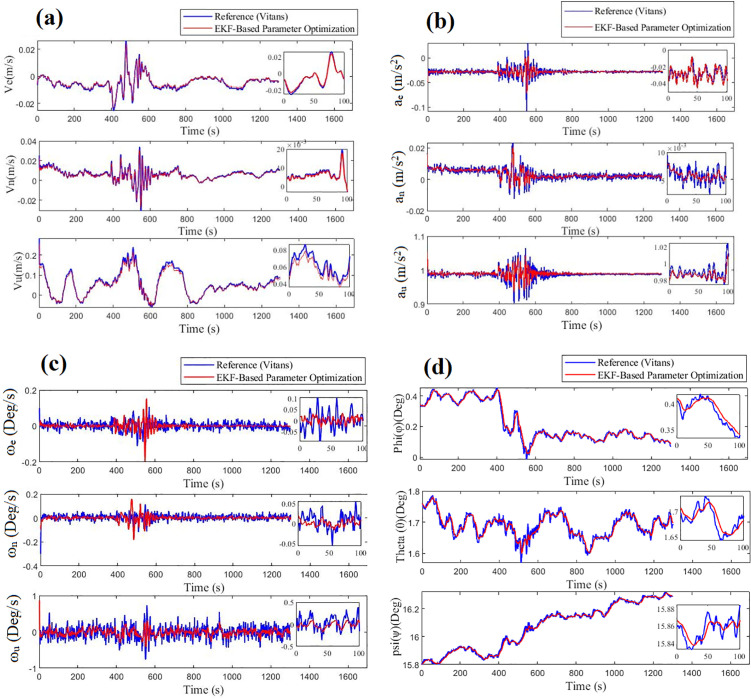
Comparison of tracking for the Vitans reference and EKF-based parameter optimization.

The results presented in Fig 9 show a comparison between the reference data (VITANS) and the optimized estimates obtained through the EKF–based parameter calibration.

Fig 9 (a)–(d) provide a comprehensive representation of the tracking performance in velocity, acceleration, angular velocity, and Euler angles along the three axes. The optimization of parameters using the EKF framework clearly results in significant improvements in both accuracy and stability. Notably, the tracking of velocity, acceleration, and angular velocity reveals a marked reduction in noise and fluctuations, leading to smoother and more precise estimates compared to reference data. This reduction in noise and the resulting higher stability can be attributed to the effective calibration and optimization of sensor parameters. For Euler angles, similar improvements were observed, with a substantial reduction in drift and better consistency in tracking rotational movements. These findings demonstrate that the EKF-based parameter optimization enhances the system’s robustness, making it less susceptible to environmental disturbances and dynamic platform noise. In practical terms, the application of EKF optimizations not only enhances the accuracy of measurements but also increases the overall reliability of the system in real-world scenarios.

**Fig 10:**
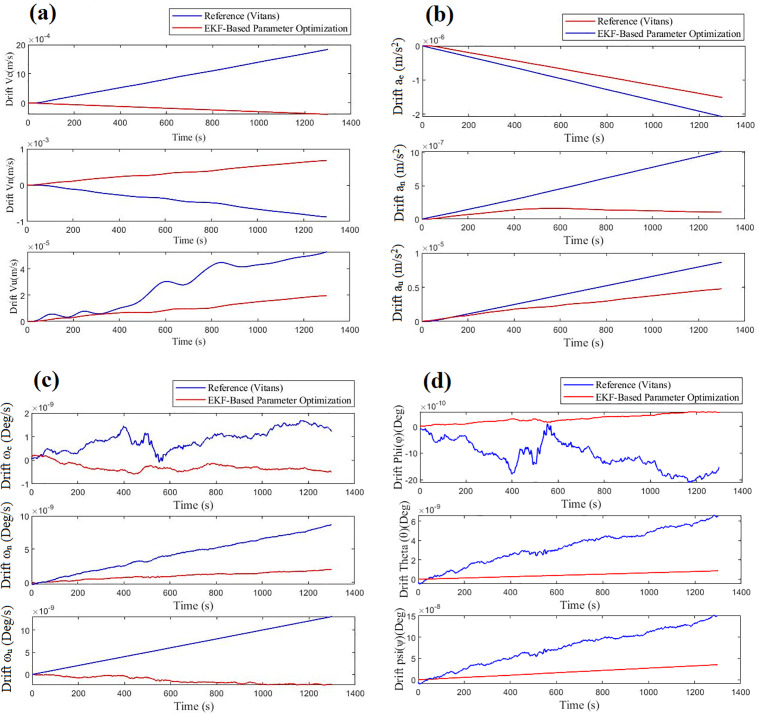
Comparison of drift for the Vitans reference and EKF-based parameter optimization.

Fig 10 (a)–(d) provide a detailed analysis of the drift in velocity, acceleration, angular velocity, and Euler angles during the experimental test. The results clearly show a significant reduction in drift after the application of parameter optimization using EKF. This reduction in drift is particularly evident over extended time periods, where the system demonstrates improved long-term stability. The EKF optimization effectively mitigates the influence of noise and non-linear variations, ensuring that the system remains accurate and stable, even under dynamic and challenging conditions. Compared to reference data, the drift in the Euler angles and angular velocity is notably reduced, demonstrating the system’s enhanced ability to maintain precision in the presence of external disturbances. These improvements highlight the system’s enhanced resilience and performance, making it more suitable for high-precision navigation and inertial sensing applications, where long-term stability is critical.

The results presented in this study demonstrate the effectiveness of the EKF-based optimization in significantly improving tracking accuracy and reducing drift in key inertial parameters, including velocity, acceleration, angular velocity, and Euler angles. The reduction in noise and fluctuations, together with the improved long-term stability, confirms the robustness of the proposed system under real-world dynamic conditions. These improvements enhance the precision of inertial navigation systems and highlight their suitability for demanding applications such as autonomous systems, aerospace, and maritime navigation. Moreover, the integration of quantum-based inertial sensors with advanced filtering techniques reinforces the effectiveness of the proposed hybrid framework in achieving high accuracy and reliability. The findings emphasize the critical role of accurate parameter calibration and advanced estimation frameworks in overcoming the limitations of conventional systems, thereby enabling next-generation inertial sensors for high-performance navigation and scientific applications.

### Raman spectroscopy analysis of rubidium

The designed calibration method of AI rate integrator sensor yields converged estimation of all calibration parameters as $k_{𝐞𝐟𝐟},\Phi_{𝐞𝐟𝐟},\Delta ,\delta_{12},C_{e},C_{g},\Omega_{e},\Omega_{g}$ in Eq. (28). As a significant result, the parameters are replaced in the wavelength model to obtain an optimal value of the desired λ of the Laser Raman Rb. Applying this λ, in the spectroscopic analysis of the Raman Laser beams generator leads to accurate characterization of fed Rb. Therefore, the optimal estimation of λ of the UNI DRON Raman spectroscopy device based on final objected navigation path purifies the sensors sensitivity. As a matter of fact, applying this λ of the Rb into the UNI DRON Raman spectroscopy device results in high accuracy spectrum analysis test related to the element Rb. It was observed that with respect to the accurate λ and corresponding wavelength frame, the intensity of Rb-related spectra became more prominent and clear. This result serves as a validation of our designed calibration method and the software in the loop test results using the experimental test data of measured trajectory by the AI rate integrator.

The test setup is organized into three main steps: labeling, Raman signal acquisition, and data analysis for the practical experimental configuration. After preparing the Rb sample and placing it in the Raman spectroscopy device, the wavelength λ identified by the EKF results is used as the main parameter of the laser beam source applied to the sample.

**Fig 11:**
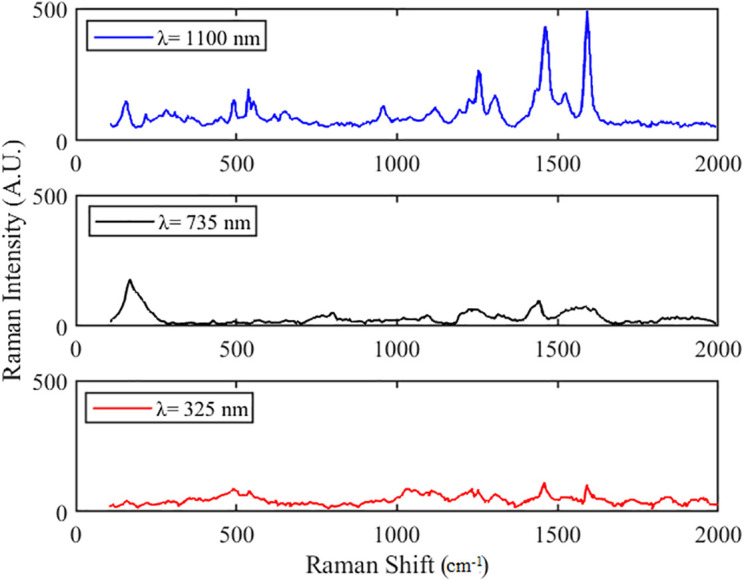
Comparison of Rubidium spectra at three wavelength 325 nm, 735 nm and 1100 nm.

The obtained Raman spectrum shows the intensity of the Raman scattering peaks for Rb film upon applying different wavelength in Fig 11. According to the spectrum-found intensity, the Raman scattering peaks for each sample at different Raman shifts (*cm*^*-1*^) appear as the difference in wavelength between the incident laser light and the scattered light.

As disclosed in Fig 11, the peaks at 325 nm, 735 nm, and 1100 nm are main characteristics of this sample. By radiating 1100 nm, the spectra in Fig 11 show higher intensity and more clearly detectable peaks compared to the 325 *nm*, 735 *nm*. These peaks are characteristic of the vibrational modes of the molecules in Rb. The resolution of the spectrum by applying 325 *nm* is not very high, so some of the smaller peaks may not be visible for detection. Furthermore, there exist are new broad peaks around 700 *cm*^*-1*^ and 1450 *cm*^*-1*^ while would not be present in the Rb spectra by 735 *nm* and 325 *nm*. These new peaks are attributed to the interaction between Rb molecules, which enhances the Raman scattering of Rb in desired λ.

In the 325 *nm* spectrum of Rb, there is no peak near 1300 *cm*^*-1*^, the rotational mode frequency, and therefore, it is believed that this mode is weakly coupled to the lowest energy mode in this orientation. This peak is weakly visible at 735 *nm*. The rotational mode was therefore shifted to 1500 *cm*^*-1*^ and the low energy mode, which is at 1250 *cm*^*-1*^ in both 325 *nm* and 735 *nm*, is correspondingly shifted up to 1500 *cm*^*-1*^ and 1600 *cm*^*-1*^. Observing the rotational mode at 1500 *cm*^*-1*^ and 1600 *cm*^*-1*^in this orientation, we see no peak at the energy by 325 *nm* and 735 *nm*. In this case, we have showed that by approaching the proper λ, peaks of the rotational mode are visible and strongly coupled to the highest energy mode that leads to increase of sensitivity.

## Conclusion

In this study, we investigate the parametric calibration of a novel AI rate integrator model. Through a comparative analysis of the sensitivity of two atomic sensor configurations, we demonstrate that the four-pulse AI model in the Butterfly configuration provides twice the sensitivity of the initial model. Consequently, its implementation in a fountain geometry leads to a twofold improvement in measurement accuracy.

Our results indicate that accurate estimation of calibration parameters using the EKF significantly enhances the overall performance of this class of rate sensors. To further validate the increased sensitivity of the AI rate integrator, an additional experimental test was performed. In this experiment, a Raman spectroscopy setup was employed to probe the Rb spectrum using photons of a specific wavelength.

The measured energy gap between the ground and excited states reveals key information about molecular bonding, enabling improved spectral resolution. This two-photon Raman process enhances spectroscopic performance and contributes to higher-precision measurements in atom interferometry applications.

## Supporting information

S1 Data S1 File. Supporting dataset and implementation code. S2 File. Appendix A.
(RAR)

## References

[1] Wright MJ, Anastassiou L, Mishra C, Davies JM, Phillips AM, Maskell S, et al. Cold atom inertial sensors for navigation applications. Front Phys. 2022;10. doi: 10.3389/fphy.2022.994459

[2] Wang X, Kealy A, Gilliam C, Haine S, Close J, Moran B, et al. Improving measurement performance via fusion of classical and quantum accelerometers. J Navigation. 2023;76(1):91–102. doi: 10.1017/s0373463322000637

[3] Saywell JC, Carey MS, Light PS, Szigeti SS, Milne AR, Gill KS, et al. Enhancing the sensitivity of atom-interferometric inertial sensors using robust control. Nat Commun. 2023;14(1):7626. doi: 10.1038/s41467-023-43374-0 37993456 PMC10665367

[4] Salducci C, Bidel Y, Cadoret M, Darmon S, Zahzam N, Bonnin A, et al. Quantum sensing of acceleration and rotation by interfering magnetically launched atoms. Sci Adv. 2024;10(44):eadq4498. doi: 10.1126/sciadv.adq4498 39475600 PMC11524193

[5] Yang J, Wang X, Nie G. A novel method for improving the long-term stability of inertial devices based on model prediction. Mechanical Systems and Signal Processing. 2025;228:112492. doi: 10.1016/j.ymssp.2025.112492

[6] Titterton D, Weston JL, Weston J. Strapdown inertial navigation technology. IET. 2004. doi: 10.1049/PBRA017E

[7] Khandakar A, Michelson DG, Naznine M, Salam A, Nahiduzzaman M, Khan KM, et al. Harnessing Smartphone Sensors for Enhanced Road Safety: A Comprehensive Dataset and Review. Sci Data. 2025;12(1):418. doi: 10.1038/s41597-024-04193-0 40064898 PMC11893768

[8] Kasevich M, Chu S. Atomic interferometry using stimulated Raman transitions. Phys Rev Lett. 1991;67(2):181–4. doi: 10.1103/PhysRevLett.67.181 10044515

[9] Jekeli C. Navigation error analysis of atom interferometer inertial sensor. NAVIG. 2005. doi: 10.1002/j.2161-4296.2005.tb01726.x

[10] Li W, Wen Z, Yang G, Zhang Y, Mo H, Peng J, et al. Calibration and Compensation of Gyro Drift Errors Based on External Rotational Angle Comparison in a Rotational Inertial Navigation System. Applied Sciences. 2025;15(3):1667. doi: 10.3390/app15031667

[11] Wei Y, Yang J, Li P, Zhang J, Liang P. Compensation Techniques for Photosensors Used in High-Precision Accelerometers. Micromachines. 2024. doi: 10.3390/mi15091131 PMC11433842 39337791

[12] Kornack TW. A test of CPT and Lorentz symmetry using a potassium-helium-3 co-magnetometer. Princeton University. 2005.

[13] Gustavson TL, Bouyer P, Kasevich MA. Precision Rotation Measurements with an Atom Interferometer Gyroscope. Phys Rev Lett. 1997;78(11):2046–9. doi: 10.1103/physrevlett.78.2046

[14] Snadden M, McGuirk J, Bouyer P, Haritos K, Kasevich M. Measurement of the Earth’s Gravity Gradient with an Atom Interferometer-Based Gravity Gradiometer. Phys Rev Lett. 1998;81(5):971–4. doi: 10.1103/physrevlett.81.971

[15] Gustavson TL, Landragin A, Kasevich MA. Rotation sensing with a dual atom-interferometer Sagnac gyroscope. Class Quantum Grav. 2000;17(12):2385–98. doi: 10.1088/0264-9381/17/12/311

[16] Peters A, Chung KY, Chu S. High-precision gravity measurements using atom interferometry. Metrologia. 2001;38(1):25–61. doi: 10.1088/0026-1394/38/1/4

[17] McGuirk JM, Foster GT, Fixler JB, Snadden MJ, Kasevich MA. Sensitive absolute-gravity gradiometry using atom interferometry. Phys Rev A. 2002;65(3). doi: 10.1103/physreva.65.033608

[18] Durfee DS, Shaham YK, Kasevich MA. Long-term stability of an area-reversible atom-interferometer Sagnac gyroscope. Phys Rev Lett. 2006;97(24):240801. doi: 10.1103/PhysRevLett.97.240801 17280264

[19] Müller H, Peters A, Chu S. A precision measurement of the gravitational redshift by the interference of matter waves. Nature. 2010;463(7283):926–9. doi: 10.1038/nature08776 20164925

[20] Abend S, Allard B, Arnold AS, Ban T, Barry L, Battelier B, et al. Technology roadmap for cold-atoms based quantum inertial sensor in space. AVS Quantum Science. 2023;5(1). doi: 10.1116/5.0098119

[21] Chen Z, Qin F, Lu S, Li R, Jiang M, Wang Y, et al. A Novel Integrated Inertial Navigation System with a Single-Axis Cold Atom Interferometer Gyroscope Based on Numerical Studies. Micromachines (Basel). 2025;16(8):905. doi: 10.3390/mi16080905 40872412 PMC12388500

[22] van Goor P, Hamel T, Mahony R. Synchronous observer design for Inertial Navigation Systems with almost-global convergence. Automatica. 2025;177:112328. doi: 10.1016/j.automatica.2025.112328

[23] d’Armagnac de Castanet Q, Des Cognets C, Arguel R, Templier S, Jarlaud V, Ménoret V, et al. Atom interferometry at arbitrary orientations and rotation rates. Nat Commun. 2024;15(1):6406. doi: 10.1038/s41467-024-50804-0 39080301 PMC11289413

[24] Tennstedt B, Rajagopalan A, Weddig NB, Abend S, Schön S, Rasel EM. Atom Strapdown: Toward Integrated Quantum Inertial Navigation Systems. navi. 2023;70(4):navi.604. doi: 10.33012/navi.604

[25] Meng Z-X, Yan P-Q, Wang S-Z, Li X-J, Xue H-B, Feng Y-Y. Closed-loop dual-atom-interferometer inertial sensor with continuous cold atomic beams. Physical Review Applied. 2024. doi: 10.1103/PhysRevApplied.21.034050

[26] HosseiniArani A, Tennstedt B, Schilling M, Knabe A, Wu H, Schön S, et al. Kalman-Filter Based Hybridization of Classic and Cold Atom Interferometry Accelerometers for Future Satellite Gravity Missions. International Association of Geodesy Symposia. Springer International Publishing. 2022. 221–31. doi: 10.1007/1345_2022_172

[27] Berman PR. Atom interferometry. Academic Press. 1997.

[28] ali maher tarek, zahran shady, Oda ESS, Nafea SF. Integrated INS/GNSS Navigation Systems: A Comprehensive Review of Filtering and AI-Based Fusion Techniques. Suez Canal Engineering, Energy and Environmental Science. 2025;3(3):17–30. doi: 10.21608/sceee.2025.378670.1076

[29] Lenef A, Hammond TD, Smith ET, Chapman MS, Rubenstein RA, Pritchard DE. Rotation Sensing with an Atom Interferometer. Phys Rev Lett. 1997;78(5):760–3. doi: 10.1103/physrevlett.78.760 10059731

[30] Storey P. and Cohen-Tannoudji C, The Feynman path integral approach to atomic interferometry. A tutorial. Journal de Physique II. 1994; 10.1051/jp2:1994103

[31] Rabitz H, de Vivie-Riedle R, Motzkus M, Kompa K. Whither the future of controlling quantum phenomena?. Science. 2000. doi: 10.1126/science.288.5467.824 10796997

[32] Gordon RJ, Rice SA. Active control of the dynamics of atoms and molecules. Annu Rev Phys Chem. 1997;48:601–41. doi: 10.1146/annurev.physchem.48.1.601 15012451

[33] Rice SA, Zhao M. Optical control of molecular dynamics. 2000.

[34] Bartels R, Backus S, Zeek E, Misoguti L, Vdovin G, Christov I, et al. Shaped-pulse optimization of coherent emission of high-harmonic soft X-rays. Nature. 2000;406(6792):164–6. doi: 10.1038/35018029 10910350

[35] Rangan C, Bucksbaum P. Optimally shaped terahertz pulses for phase retrieval in a Rydberg-atom data register. Physical Review A. 2001. doi: 10.1103/PhysRevA.64.033417

[36] Canuel B, Leduc F, Holleville D, Gauguet A, Fils J, Virdis A, et al. Six-axis inertial sensor using cold-atom interferometry. Phys Rev Lett. 2006;97(1):010402. doi: 10.1103/PhysRevLett.97.010402 16907358

[37] Fang J, Wan S, Yuan H. Dynamics of an all-optical atomic spin gyroscope. Appl Opt. 2013;52(30):7220–7. doi: 10.1364/AO.52.007220 24216575

[38] Gauguet A, Canuel B, Lévèque T, Chaibi W, Landragin A. Characterization and limits of a cold-atom Sagnac interferometer. Phys Rev A. 2009;80(6). doi: 10.1103/physreva.80.063604

[39] Tackmann G, Berg P, Abend S, Schubert C, Ertmer W, Rasel EM. Large-area Sagnac atom interferometer with robust phase read out. Comptes Rendus Physique. 2014;15(10):884–97. doi: 10.1016/j.crhy.2014.10.001

[40] Gustavson TL. Precision rotation sensing using atom interferometry. Stanford University. 2000.

